# Ventilation and perfusion MRI at a 0.35 T MR-Linac: feasibility and reproducibility study

**DOI:** 10.1186/s13014-023-02244-1

**Published:** 2023-04-03

**Authors:** Rabea Klaar, Moritz Rabe, Thomas Gaass, Moritz J. Schneider, Ilyes Benlala, Chukwuka Eze, Stefanie Corradini, Claus Belka, Guillaume Landry, Christopher Kurz, Julien Dinkel

**Affiliations:** 1 Department of Radiology, University Hospital, LMU Munich, Munich, Germany; 2 Comprehensive Pneumology Center (CPC-M), Member of the German Center for Lung Research (DZL), Munich, Germany; 3 Department of Radiation Oncology, University Hospital, LMU Munich, Munich, Germany; 4 Antaros Medical AB, BioVenture Hub, Mölndal, Sweden; 5 Univ. Bordeaux, Centre de Recherche Cardio-thoracique de Bordeaux, F-33600 Pessac, France; 6 CHU Bordeaux, Service d’Imagerie Thoracique et Cardiovasculaire, Service des Maladies Respiratoires, Service d’Exploration Fonctionnelle Respiratoire, Unité de Pneumologie Pédiatrique, CIC 1401, F-33600 Pessac, France; 7 INSERM, U1045, Centre de Recherche Cardio-thoracique de Bordeaux, F-33600 Pessac, France; 8 German Cancer Consortium (DKTK), Munich, Germany

**Keywords:** Functional lung MRI, Radiation therapy, MR-Linac, Non-uniform Fourier decomposition, Ventilation, Perfusion, Low-field MRI

## Abstract

**Background:**

Hybrid devices that combine radiation therapy and MR-imaging have been introduced in the clinical routine for the treatment of lung cancer. This opened up not only possibilities in terms of accurate tumor tracking, dose delivery and adapted treatment planning, but also functional lung imaging. The aim of this study was to show the feasibility of Non-uniform Fourier Decomposition (NuFD) MRI at a 0.35 T MR-Linac as a potential treatment response assessment tool, and propose two signal normalization strategies for enhancing the reproducibility of the results.

**Methods:**

Ten healthy volunteers (median age 28 ± 8 years, five female, five male) were repeatedly scanned at a 0.35 T MR-Linac using an optimized 2D+*t* balanced steady-state free precession (bSSFP) sequence for two coronal slice positions. Image series were acquired in normal free breathing with breaks inside and outside the scanner as well as deep and shallow breathing. Ventilation- and perfusion-weighted maps were generated for each image series using NuFD. For intra-volunteer ventilation map reproducibility, a normalization factor was defined based on the linear correlation of the ventilation signal and diaphragm position of each scan as well as the diaphragm motion amplitude of a reference scan. This allowed for the correction of signal dependency on the diaphragm motion amplitude, which varies with breathing patterns. The second strategy, which can be used for ventilation and perfusion, eliminates the dependency on the signal amplitude by normalizing the ventilation/perfusion maps with the average ventilation/perfusion signal within a selected region-of-interest (ROI). The position and size dependency of this ROI was analyzed. To evaluate the performance of both approaches, the normalized ventilation/perfusion-weighted maps were compared and the deviation of the mean ventilation/perfusion signal from the reference was calculated for each scan. Wilcoxon signed-rank tests were performed to test whether the normalization methods can significantly improve the reproducibility of the ventilation/perfusion maps.

**Results:**

The ventilation- and perfusion-weighted maps generated with the NuFD algorithm demonstrated a mostly homogenous distribution of signal intensity as expected for healthy volunteers regardless of the breathing maneuver and slice position. Evaluation of the ROI’s size and position dependency showed small differences in the performance. Applying both normalization strategies improved the reproducibility of the ventilation by reducing the median deviation of all scans to 9.1%, 5.7% and 8.6% for the diaphragm-based, the best and worst performing ROI-based normalization, respectively, compared to 29.5% for the non-normalized scans. The significance of this improvement was confirmed by the Wilcoxon signed rank test with $p\, <\, 0.01$ at $\alpha \, =\, 0.05$. A comparison of the techniques against each other revealed a significant difference in the performance between best ROI-based normalization and worst ROI ($p\, =\, 0.01$) and between best ROI-based normalization and scaling factor ($p\, =\, 0.02$), but not between scaling factor and worst ROI ($p\, =\, 0.71$). Using the ROI-based approach for the perfusion-maps, the uncorrected deviation of 10.2% was reduced to 5.3%, which was shown to be significant ($p\, <\, 0.01$).

**Conclusions:**

Using NuFD for non-contrast enhanced functional lung MRI at a 0.35 T MR-Linac is feasible and produces plausible ventilation- and perfusion-weighted maps for volunteers without history of chronic pulmonary diseases utilizing different breathing patterns. The reproducibility of the results in repeated scans significantly benefits from the introduction of the two normalization strategies, making NuFD a potential candidate for fast and robust early treatment response assessment of lung cancer patients during MR-guided radiotherapy.

**Supplementary Information:**

The online version contains supplementary material available at 10.1186/s13014-023-02244-1.

## Background

Lung cancer is one of the leading causes of cancer related deaths worldwide [1]. Radiotherapy, and in particular adaptive radiotherapy (ART), have become more and more important in the treatment of lung cancer patients, since ART allows adaption of the treatment plan for possible anatomical and physiological changes based on computed tomography (CT) or magnetic resonance imaging (MRI) between treatment fractions [2–6]. The recent introduction in the clinical routine of hybrid systems that combine a MRI-scanner and a medical linear accelerator (MR-Linacs) allows daily ART and image-guidance [7–11]. The excellent soft-tissue contrast of MRI allows for an improved delineation of organs at risk as well as target volumes and additionally enables precise tumor-tracking and beam-gating based on cine-MRI to mitigate intra-fractional motion, resulting in dosimetric benefits [7, 12–15]. Along with being a non-invasive alternative to CT in terms of treatment planning as well as providing image-guidance during radiotherapy, MR-Linacs also enable MRI-specific methods such as functional imaging of head and neck cancer [16–19], but also functional imaging of the lung. Due to fractionated dose delivery, MR-Linacs even allow longitudinal functional data acquisition within the course of the patients’ treatment, which is especially valuable since it may permit early treatment response assessments [20–23].

For functional lung imaging, several techniques have been developed over the years. Some of these approaches require the inhalation of gases such as hyperpolarized helium ($^{3}\textrm{He}$) [24], xenon ($^{129}\textrm{Xe}$) [25], fluorine ($^{19}\textrm{F}$) [26] or oxygen [27] to assess lung ventilation, or the injection of gadolinium-based contrast agents to evaluate perfusion [28], which is not only costly but also technically challenging [29]. An alternative are Fourier Decomposition (FD) MRI [30] techniques, which are performed in free breathing and make use of the intrinsic lung signal variation due to breathing and blood flow, such as Non-uniform Fourier Decomposition (NuFD) [31], PREFUL [32] or SENCEFUL [33]. These techniques do not require a contrast agent, any special equipment or respiratory triggering and are therefore fast, easily applicable and have shown promising results in chronic thromboembolic pulmonary hypertension, asthma, chronic obstructive pulmonary disease (COPD) and cystic fibrosis (CF) studies [31, 32, 34–36]. Due to FD-MRI’s dependency on changes in the breathing pattern as well as the residual lung volume, variations in breathing amplitude from fraction to fraction may influence ventilation maps and mask pathological changes. NuFD, a robust FD-MRI technique, has been designed to correct for variations in respiratory and cardiac frequencies during the course of image acquisition by retrospectively converting equidistant sampling into non-equidistant sampling in order to track the main frequencies [31]. Their ease of applicability make FD-MRI techniques particularly well suited for longitudinal studies embedded in an MR-Linac radiotherapy workflow. However, reproducibility of the ventilation maps in such studies remains challenging and additionally requires a form of signal normalization [37]. Otherwise, focal longitudinal changes might be masked by global changes due to variations in the breathing amplitude.

Even though the aforementioned functional lung imaging methods have been developed and optimized for high-field MRI (1.5–3 T), studies by Campbell-Washburn et al. [38] and Deimling et al. [39] showed that lung imaging can benefit from lower magnetic field strengths since the susceptibility artefacts caused by local inhomogeneities at the multiple air-tissue interfaces of the lung parenchyma are reduced [40]. The resulting improved image quality suggests that the transfer and optimization of FD-MRI sequences [30] to a 0.35 T MR-Linac is desirable. So far, these methods have not been evaluated at these devices.

The aim of this study was to test the feasibility of non-contrast enhanced ventilation and perfusion MRI using NuFD at a 0.35 T MR-Linac, and to improve the reproducibility by introducing normalization strategies.

## Methods

In order to improve the reproducibility within a longitudinal study, as required for early response assessment in MR-guided radiotherapy, two ventilation normalization strategies are proposed and their performance is evaluated in a study with ten healthy volunteers. Additionally, the reproducibility of the perfusion is investigated with and without one of the introduced normalization approaches.

### Image acquisition

Ten healthy volunteers (24–52 years old, five female and five male) were scanned at a $0.35\,\textrm{T}$ MR-Linac (MRidian, Viewray Inc., Cleveland, Ohio) using a 2D balanced steady-state free precession (bSSFP) sequence that was optimized to achieve the required temporal resolution in order to observe signal intensity changes introduced by respiration and perfusion. Two coronal slice positions were selected with a field-of-view (FOV) of $500\times 500\,\textrm{mm}^{2}$, a pixel size of $3.91\times 3.91\,\textrm{mm}^{2}$, a slice thickness of $20\,\textrm{mm}$ and a matrix size of $128\times 128$. With a repetition time (TR) of $2.42\,\textrm{ms}$ and echo time (TE) of $1.02\,\textrm{ms}$, a temporal resolution of $310\,\mathrm {ms/image}$ was reached, resulting in a total acquisition time of $1.1\,\textrm{min}$ for a series of 240 images. The flip angle was $70.0^{\circ }$ and the receiver bandwidth $710.0\,\mathrm {Hz/pixel}$. The slice positions were chosen for each volunteer individually based on a 3D-bSSFP MRI-scan performed in inspiration breath-hold with a total acquisition time of $25\,\textrm{s}$. The imaging parameters were: $\textrm{TR} = 3.0\,\textrm{ms}$, $\textrm{TE} = 1.27\,\textrm{ms}$, FOV = $540\times 465\times 432\, \mathrm {mm^{3}}$, matrix = $360\times 310\times 144$, voxel size = $1.5\times 1.5\times 3.0\,\mathrm {mm^{3}}$, flip angle = $60.0^{\circ }$, receiver bandwidth = $604.0\,\mathrm {Hz/pixel}$. The vendor’s 6-channel torso coils were used to receive the MR-signal. One slice position was selected to intersect the aorta, while the other was positioned anterior or posterior of the first slice depending on the lung volume of each volunteer. In the following, the two slice positions are referred to as ’aorta’ and ’lung’. The aorta slice was selected in order to have a comparable position for all volunteers. Considering the potential application in lung cancer patients with different tumor positions and overall lung anatomy, the performance of the methods needed to be investigated at different locations within the lung, thus justifying the additional lung slice. The position was chosen to cover a large variety of slices among the volunteers, showing different parts of the lung. For volunteers with large lung volumes, the lung slice was positioned posterior to the aorta slice, while for volunteers with smaller lung volumes a slice position anterior to the aorta was selected. In order to test reproducibility, both slice positions were scanned repeatedly and with different breathing patterns for each volunteer. After acquiring the image series once for each slice in normal free breathing, a break of about $15\,\textrm{min}$ was taken inside the scanner before repeating the acquisition. To evaluate the robustness for different breathing patterns, the volunteers were then asked to breathe approximately 25% deeper. This instruction was only given to yield a realistic deeper breathing pattern. The actual diaphragm amplitude was not critical for this study. The same procedure was performed after a $15{-}20\,\textrm{min}$ break outside the scanner, albeit with a reduced in-scanner break between the first and the second regular breathing scans, which was about $2{-}5\,\textrm{min}$. The second irregular breathing scan was acquired in shallower breathing. The first in-scanner break was chosen longer to allow the volunteers to get fully accustomed in the scanner and minimize anxiety related effects in the second scan. To limit the overall acquisition time to one hour, the second in-scanner break was shortened. In order to ensure a similar volunteer position after the outside-scanner break some precautions were taken during the first positioning. Pieces of tape were fixed to the volunteers’ arms and the scanner table to mark the craniocaudal position and the relative position between volunteer and table based on the integrated laser positioning system. The x-, y- and z-coordinates of the scanner table in iso-center position were noted. For the setup after the break outside the scanner, the relative position between volunteer and table was found using the tape and again the laser system. The table was then moved to the exact coordinates used for the first acquisition. The scanning protocol and the used scan abbreviations are summarized in Table 1.

**Table 1:** The scanning protocol for the volunteers, breathing patterns and used abbreviations (NB = normal breathing, IB = irregular breathing, aB = after Break) for each scan of the corresponding slice position

Scan	Slice	Breathing pattern	Abbreviation
1	3D Volume	Breath-hold	3D-scan
2	Aorta	Normal	Reference
3	Lung		
$\sim$ 15 min break inside the scanner			
4	Aorta	Normal	NB 2
5	Lung		
6	Aorta	$\sim$ 25% deeper	IB
7	Lung		
$\sim$ 10–20 min break outside the scanner			
8	Aorta	Normal	NB aB 1
9	Lung		
$\sim$ 2–5 min break inside the scanner			
10	Aorta	Normal	NB aB 2
11	Lung		
12	Aorta	Shallow	IB aB
13	Lung		

### Image processing workflow

**Fig. 1:**
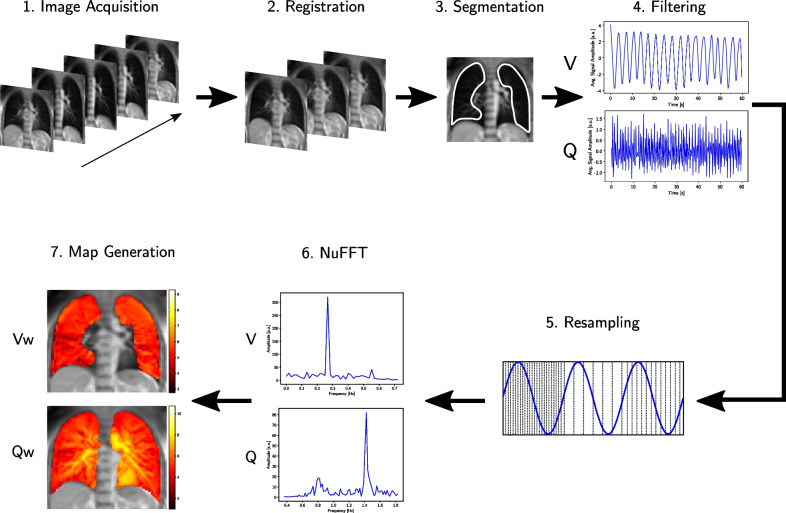
Sketch of the image processing workflow. The workflow of the NuFD consists of the acquisition of the image series in free breathing (step 1), the image registration using ANTs (step 2) and a manual segmentation (step 3). The lung signal is low- and high-pass filtered to separate the ventilation (V) and perfusion signals (Q) (step 4), respectively. Resampling based on a short-term Fourier transform is performed on both signal components individually in order to transform uniformly sampled signals with varying frequency to non-uniformly sampled signals with constant frequency (step 5). Calculating the NuFFT pixel-wise for both ventilation and perfusion (step 6) and extracting the signal amplitude of the corresponding peak allows to generate V- and Q-weighted maps of the segmented lung that are then overlayed on the original image (step 7)

The in-house developed image processing workflow was fully implemented in Python (version 3.9). As shown by Bieri and Scheffler [41], the magnetization in bSSFP sequences approaches a steady-state after several TR periods. Similar to Bondesson et al. [31], the first 20 images were discarded as the steady state condition was not fulfilled. The acquired image series were firstly aligned with a deformable image registration using ANTs (Advanced Normalization Tools) [42] to a reference image in mid-position between full inspiration and full expiration using mutual information as optimization metric employing a three-level multiresolution registration strategy (25%, 50% and 100% of the original resolution). The reference image was automatically determined within the processing workflow. For this, the overall mean signal intensity was calculated for each image as well as the temporal average over these mean signal intensities. The image closest to this average was defined as the reference image and represents a motion state close to the mid-position. Based on this reference image, a manual segmentation of the lung was performed under the supervision of an experienced radiologist. The average temporal lung signal was used to determine the subject-specific cut-off frequency between $0.55{-}1.0\,\textrm{Hz}$ to separate the ventilation and perfusion signals with a low- and high-pass Butterworth filter, respectively, which was applied forward and backwards to avoid the introduction of a phase shift. Since the Butterworth filter provides a good compromise between attenuation and phase response [43], it finds application in the processing of biomedical signals and was chosen in this study [44]. As shown by Bondesson et al. [31], a uniformly sampled signal with varying frequency is transformable into a non-uniformly sampled signal with constant frequency by defining virtual sampling times $\tilde{t}_{n}$ based on the instantaneous frequency. For this, a short-term Fourier transform was calculated and an edge-extraction algorithm (ssqueezepy package [45]) was applied to the 2D time-frequency representation to determine the instantaneous frequency of the ventilation and the perfusion signal and thus the respective virtual non-equidistant sampling times. These sampling times were then used to calculate the type-1 Non-uniform fast Fourier Transform (NuFFT) per pixel on the segmented lung. The ventilation- (Vw) and perfusion-weighted (Qw) maps were then generated by taking the maximum magnitude of the corresponding peak in the Fourier spectrum. The Vw- and Qw-maps are not quantitative but reflect the regional tissue density oscillation of the lung parenchyma due to ventilation in the former and the regional MR-signal intensity oscillation due to perfusion in the latter case. Diseased lung areas with altered parenchymal density, reduced pulmonary ventilation and/or poor perfusion would show less signal intensity in the Vw- and Qw-maps [30, 31]. The whole image processing workflow is illustrated in Fig. 1.

### Normalization strategies

#### Diaphragm amplitude scaling factor

**Fig. 2:**
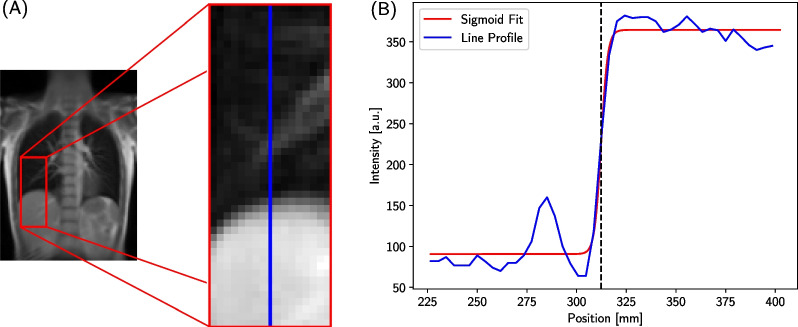
Working principle of the diaphragm position extraction. The diaphragm positions used for the scaling factor-based normalization are extracted by first selecting a ROI (**A**) to get the line profile of the intensity for each frame in the image series. Each line profile (blue) is then fitted using a sigmoid function (red) as shown in (**B**). To determine the actual position of the diaphragm, the maximum of the derivative of the fitted sigmoid function is calculated, which is indicated by the black dashed line

The idea of this normalization strategy is to introduce a multiplicative factor to normalize a scan acquired at a certain time point to a reference scan. With this, differences between scans due to breathing amplitude changes are compensated and the comparability within a longitudinal study improved. In order to correct for inter-scan differences in the Vw-maps due to variations in breathing amplitude, the relationship between lung ventilation signal and diaphragm position can be exploited. Relative changes in the average lung ventilation signal correspond to relative changes in lung volume and thus to the diaphragm motion [30]. The frame-wise lung ventilation signal is therefore normalized by the diaphragm position in this approach. The position of the diaphragm for each image frame was determined by placing a ROI around the diaphragm of the right lung and extracting the line profile along a vertical line through the diaphragm, as shown in Fig. 2A. Each of these line profiles was then fitted with a sigmoid function. The derivative of the sigmoid function was computed and its maximum position, i.e., the maximal intensity change, was used to determine the position of the diaphragm (Fig. 2B). Relative diaphragm positions were calculated with respect to the intermediate state. According to the definition used in this study, positive position values correspond to inspiration and negative values to expiration.

**Fig. 3:**
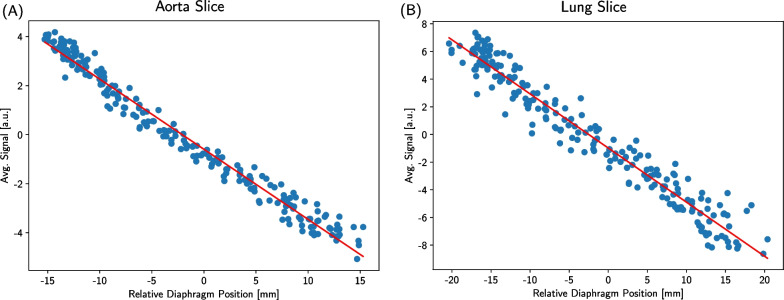
The linear diaphragm position and lung signal correlation. The correlation between the relative diaphragm position and the average lung ventilation signal of each frame are exemplarily shown for a scan of the aorta (**A**) and lung slice (**B**). As a reference for the diaphragm positions, the mid position between full inspiration and full expiration was used, meaning that positive values describe the increase in lung volume and consequently negative values the decrease in lung volume. Regardless of the considered slice, a linear relationship between the two quantities is observable. The slope of the linear fit function depicted in red finds application in Eq. 1

Correlating these diaphragm positions with the corresponding average lung ventilation signal in each image revealed a linear relationship. This allows to fit the correlation and to extract the slope $\textrm{d}S/\textrm{d}x$ with the filtered lung ventilation signal *S* (step 4 in Fig. 1) and the relative diaphragm position *x*. This is examplarily shown for both slice positions of Volunteer 5 in Fig. 3. More examples of the correlation for different volunteers and scans can be found in the Additional File 1. This factor for a scan *i* is defined by:

1 $$\begin{aligned} \text {normalization factor}_{i} = \dfrac{\textrm{d}S_{i}}{\textrm{d}x_{i}} \cdot \dfrac{\overline{x}_{\text {ref,max}} - \overline{x}_{\text {ref,min}}}{\overline{S}_{i,\text {max}} - \overline{S}_{i,\text {min}}}, \end{aligned}$$

where $\textrm{d}S_{i}/\textrm{d}x_{i}$ is the slope of the linear fit and $\overline{S}_{i,\text {max}}$ and $\overline{S}_{i,\text {min}}$ are the mean maxima and minima of the filtered lung ventilation signal of scan *i*. The mean maxima and minima of the relative diaphragm positions in the reference scan are $\overline{x}_{\text {ref,max}}$ and $\overline{x}_{\text {ref,min}}$, respectively. A visual explanation of the parameters used in Eq. 1 and their extraction is given in the Additional File 2. Multiplying this resulting factor to the ventilation signal before the pixel-wise NuFFT (step 6 in Fig. 1) allows to correct for differences in the diaphragm amplitude between scan *i* and the reference scan.

#### Region-of-interest normalization

**Fig. 4:**
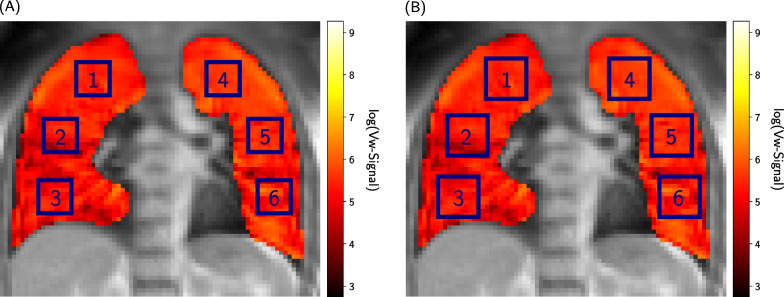
The small and large ROI positions. The positions and numbering of the ROIs, whose mean ventilation value is used for the normalization of the ventilation-weighted maps, are displayed. In **A** the small ROIs with a size of $8\times 8$ pixels and in **B** the large ROIs with $12\times 12$ pixels are shown in blue with their assigned numbers starting from the top right lung. For presentation purposes, the ventilation maps were filtered using a Gaussian and then logarithmically plotted

An alternative approach for the normalization of consecutive scans is to normalize the Vw- and Qw-maps of each scan pixel-wise by the average value within a chosen ROI where the lung parenchyma is assumed to be healthy. Assuming that breathing pattern changes affect all parts of the lung in a similar manner, this strategy diminishes the dependence on the breathing amplitude. The final normalized maps $\Gamma _{\text {norm}}$ are then given by:

2 $$\begin{aligned} \Gamma _{\text {norm}} = \dfrac{\Gamma }{\overline{\Gamma }\!\left( \text {ROI}\right) } \end{aligned}$$

with $\Gamma = \textrm{Vw, Qw}$ the uncorrected maps and $\overline{\Gamma }\!\left( \text {ROI}\right)$ the mean map value within the selected ROI of the same scan. In order to analyze the possible spatial and size dependence of the chosen region used for the normalization, six different positions (three in each lung), as shown in Fig. 4, were evaluated for two different square ROI sizes of $8\times 8$ pixels (Fig. 4A) and $12\times 12$ pixels (Fig. 4B).

### Evaluation method

#### Map comparison

The Vw- and Qw-maps show only the relative signal differences within the lung and are therefore not quantitative. Thus, the aim of the normalization was to get Vw- and Qw-maps of similar intensity despite changes in the underlying breathing pattern and other potential inter-scan differences. In order to quantify the similarity, the mean value segmented lung was calculated for the maps of each scan and compared to the corresponding reference scan map. For healthy volunteers we assume that there should be no change in the maps from scan to scan. The absolute relative deviation $\delta _{\Gamma }$ of the mean value between unnormalized/normalized maps $\overline{\Gamma }_{j}$ of scans *j* and the map of the reference scan ($\overline{\Gamma }_{\text {ref}}$) is defined as:

3 $$\begin{aligned} \delta _{\Gamma } = \left| \dfrac{\overline{\Gamma }_{\text {ref}} - \overline{\Gamma }_{j}}{\overline{\Gamma }_{\text {ref}}} \right| \end{aligned}$$

with *j* being a non-reference scan. In the following analysis, the firstly acquired aorta and lung image series served as the respective reference scans.

#### Statistical analysis

Since there is no reason to assume that the deviations of each scan’s map from the corresponding reference are normally distributed for any normalization method, the statistical evaluation for significant differences between the ventilation normalization methods was performed using a Wilcoxon signed rank test (scipy.stats.wilcoxon package; version 1.7.2). Although the Vw- and Qw-map deviations can be positive as well as negative, due to over- or underestimation by the normalization techniques, only the absolute deviations were considered. This was to evaluate the performance of the methods solely in terms of magnitude of the deviation rather than direction. For this, each approach was compared to the maps of the uncorrected scans and against each other for both slices separately and the combined total of $n=96$ scans at $\alpha = 0.05$.

## Results

### Feasibility

**Fig. 5:**
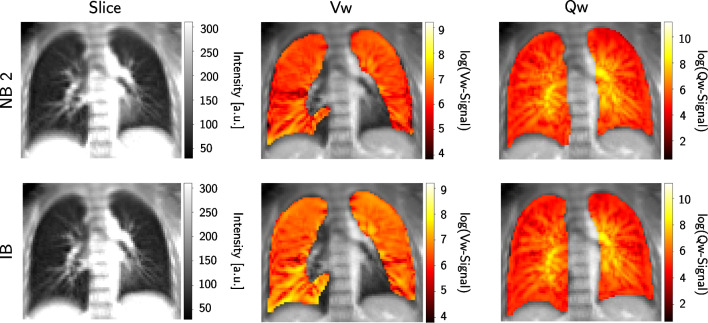
Exemplary ventilation-(Vw) and perfusion-weighted (Qw) maps. The unnormalized V- and Q-maps for the normal (NB 2) and the first irregular breathing scan (IB, deep breathing) exemplarily presented for the aorta slice of Volunteer 4. The heart and large vessels were excluded from the segmentation of the Vw-maps. The maps were Gaussian filtered and logarithmically plotted

Figure 5 displays Vw- and Qw-maps along with the 2D MR image for two exemplary volunteer scans in normal and deep breathing without normalization. Large vessels and the heart in the lower left and right lung were excluded from the segmented lung used to calculate the Vw-maps. The maps show an overall homogenous intensity which is increased in the vessels for the Qw-maps. Using the same window for the Vw-maps in normal and deep breathing results in differences in the overall intensity, as expected. Since the Qw intensity is mostly independent of the breathing pattern, the Qw-maps in Fig. 5 show no noticeable difference.

### ROI size and position analysis

The ROI position and size dependency was investigated for both the Qw-maps and Vw-maps by using the ROIs specified in Fig. 4.

#### Ventilation

The median $\delta _{V}$ of all volunteers for each ROI location and slice position as well as the combined scans are listed in Table 2. Due to inconsistencies in the scanning protocol and the instructions given to Volunteer 1 resulting in unrealistic breathing patterns, both irregular breathing scans had to be excluded from the analysis. The boxplots showing the signed deviations for the small and large ROIs using the maps of all scans regardless of slice position can be found in the Additional File 3. For the small ROIs, the smallest and largest $\delta _{V}$ for both slice positions was achieved for ROI 5 (middle left lung) and ROI 4 (top left lung), respectively. These positions also coincide with the best and worst ROI positions found for the combined maps of both slice positions.

For the large ROIs, the best results were obtained using ROI 3 (bottom right lung) for the aorta slice and ROI 5 for the lung slice. The worst ROI was found to be ROI 4 (top left lung) for the aorta slice and ROI 1 (top right lung) for the lung slice. Combining the maps of both slice positions, ROI 6 and ROI 4 result in the smallest and largest deviations, respectively. Since the large ROIs presents overall smaller deviations compared to the small ROIs, the best (ROI 6) and worst (ROI 4) of the large ROIs were considered for further analysis of the ventilation.

**Table 2:** The median values of the absolute deviations $\delta _{V}$ between each Vw-map mean and the reference presented here depending on the ROI location, the size and the slice position. The median deviations for the combined scans are also listed. All values are given in %

			Median $\varvec{\delta }_{\bf V}$ over volunteers in %											
Uncorrected			ROI 1		ROI 2		ROI 3		ROI 4		ROI 5		ROI 6	
Aorta	Lung		Aorta	Lung	Aorta	Lung	Aorta	Lung	Aorta	Lung	Aorta	Lung	Aorta	Lung
24.9	35.6	Small	5.8	11.0	6.0	9.0	8.1	10.8	8.8	12.3	5.4	7.0	8.5	7.5
		Large	6.0	10.1	6.6	8.6	5.0	7.5	7.7	9.8	7.5	5.4	5.7	6.8

Median of aorta and lung slices														
29.5		Small	8.3		7.7		9.3		9.3		6.4		8.1	
		Large	7.4		7.0		6.5		8.6		6.6		5.7	

#### Perfusion

In Table 3, the median $\delta _{Q}$ values of all volunteers for each ROI position and size are shown for both slice positions as well as the combined scans. While the uncorrected scans demonstrated

deviations below 10 %, in the case of the aorta slice, normalization using all ROI positions except ROI 1 and ROI 4 leads to further reduced deviations. The smallest $\delta _{Q}$ for the aorta slice was found for ROI 3 (small ROI) and ROI 5 (large ROI). For the lung slice, only normalization using ROI 6 for the small squares and ROI 5 for the large squares achieved an improvement in $\delta _{Q}$.

Normalization with ROI 6 for the small squares and ROI 5 for the large squares provided the best performance for the combination of all scans. Similar to the Vw-maps, the large ROIs lead to overall better results and were therefore considered for further analysis. Since using no correction showed better results than using the worst ROI (ROI 1), only ROI 5 as the best perfusion ROI was taken into account.

**Table 3:** The median values of the absolute deviations $\delta _{Q}$ of all volunteers Qw-maps for each ROI size and location as well as slice position. The median deviations for the combined scans are also listed. All values are given in %

			Median $\varvec{\delta }_{\bf Q}$ over volunteers in %											
Uncorrected			ROI 1		ROI 2		ROI 3		ROI 4		ROI 5		ROI 6	
Aorta	Lung		Aorta	Lung	Aorta	Lung	Aorta	Lung	Aorta	Lung	Aorta	Lung	Aorta	Lung
11.0	8.8	Small	12.3	11.4	8.9	9.3	6.2	12.5	9.7	14.4	7.5	9.7	10.8	6.2
		Large	12.1	11.2	5.9	11.7	6.3	8.8	11.4	11.2	4.9	5.7	6.9	10.4

Median of aorta and lung slices														
10.2		Small	11.9		9.0		9.7		13.1		8.2		7.6	
		Large	11.6		7.6		8.2		11.3		5.3		7.9	

### Reproducibility

#### Ventilation

**Fig. 6:**
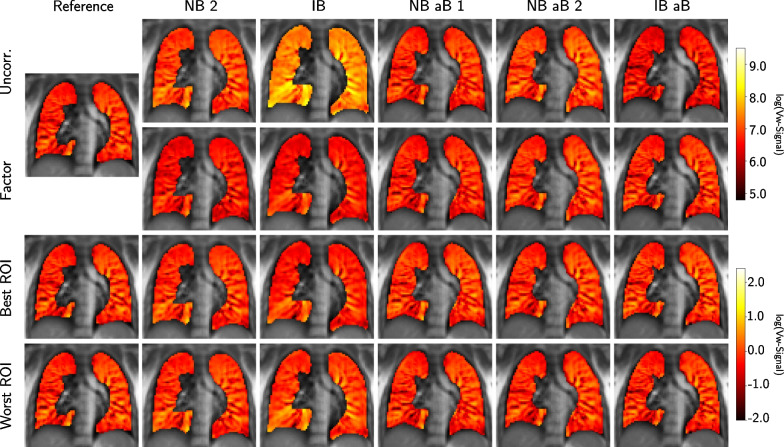
Comparison of uncorrected and normalized Vw-maps. The performance comparison between the scaling factor, the best and worst ROI-based normalization technique and the uncorrected scans exemplarily illustrated for Vw-maps of the aorta slice scans of Volunteer 5

The maps of the scans performed with different breathing patterns were each normalized using the diaphragm scaling factor and the ROI normalization strategy. In Fig. 6, the uncorrected and all corrected Vw-maps from both normalization strategies are shown for the selected aorta slice of Volunteer 5. As mentioned above, the best and worst ROI positions were considered for the ventilation analysis and therefore both are presented in Fig. 6. Looking at the Vw-maps of the uncorrected scans, especially IB and IB aB look noticeably different from the reference Vw-map when using the same window for the color map. The Vw-maps of IB (deep breathing) and IB aB (shallow breathing) display the expected higher and lower intensities, respectively, due to the specified breathing patterns.

Additionally, slight intensity differences are observable between the reference map and the Vw-maps of NB 2 and NB aB 2. These differences and especially the strongly increased signal intensity in the IB Vw-map are clearly reduced by the diaphragm-based scaling factor. Only a small underestimation is visually detectable for Volunteer 5 using this approach. Since the ROI-based approach also requires to normalize the reference map in order to validate the similarity of each map, a direct comparison to the uncorrected maps is not possible. All scans normalized with this method present only small differences in the Vw-maps. A slight overestimation of signal intensity is visible in the bottom right and middle left lung of the IB Vw-map normalized using the worst ROI position, whereas a minor underestimation of the whole lung can be noted using the best ROI position. Besides these observations, no other distinct differences in the performance between the best and the worst ROI are discernible.

Apart from the visual evaluation, the resulting normalized Vw-maps were also quantitatively analyzed per volunteer and between the volunteers. The mean ventilation was calculated for each map and compared to the reference map by determining $\delta _{V}$ from Eq. 3. The $\delta _{V}$ values for each Vw-map of Volunteer 5, as shown in Fig. 6, can be found in the Additional File 4. The median $\delta _{V}$ in % for each slice position, volunteer and normalization method are listed in Table 4. Here, as described before, only the best and worst ROIs were considered. Except for Volunteer 1 (only normal breathing scans) using the normalization factor and the worst ROI, the aorta slice of Volunteer 2 using the worst ROI, the lung slice of Volunteer 4 using the normalization factor and the worst ROI, and the lung slice of Volunteer 9 using the best ROI, all deviations calculated for the normalized Vw-maps are clearly reduced compared to the deviations for the uncorrected maps.

**Table 4:** The median $\delta _{V}$ values are listed in % for each volunteer and slice position using no correction, the normalization factor and the large ROIs 6 (best) and 4 (worst) for the ROI-based normalization

	Median $\varvec{\delta }_{V}$ over scans in %							
Volunteers	Uncorrected		Normalization factor		Best large ROI		Worst large ROI	
	Aorta	Lung	Aorta	Lung	Aorta	Lung	Aorta	Lung
Vol 1	12.8	34.9	16.5	21.7	9.3	16.4	16.1	34.0
Vol 2	16.8	23.1	11.6	3.7	10.3	9.3	29.6	11.7
Vol 3	45.5	37.8	16.7	7.4	5.7	4.4	7.5	3.4
Vol 4	17.0	6.2	3.2	14.1	13.2	3.9	12.9	7.9
Vol 5	23.6	44.2	9.7	4.6	5.0	4.4	5.1	12.2
Vol 6	36.5	52.7	13.3	11.4	3.3	2.8	6.7	8.6
Vol 7	26.9	17.9	1.5	4.0	1.8	3.7	22.0	12.4
Vol 8	38.2	50.0	8.1	7.1	6.0	13.4	2.8	5.6
Vol 9	22.7	18.1	12.0	11.5	2.3	22.0	10.6	11.6
Vol 10	25.7	29.5	7.6	13.6	6.5	8.4	5.0	13.6
Median	24.9	35.6	9.1	9.5	5.7	6.8	7.5	9.8
All Scans Median	29.5		9.1		5.7		8.6	

**Fig. 7:**
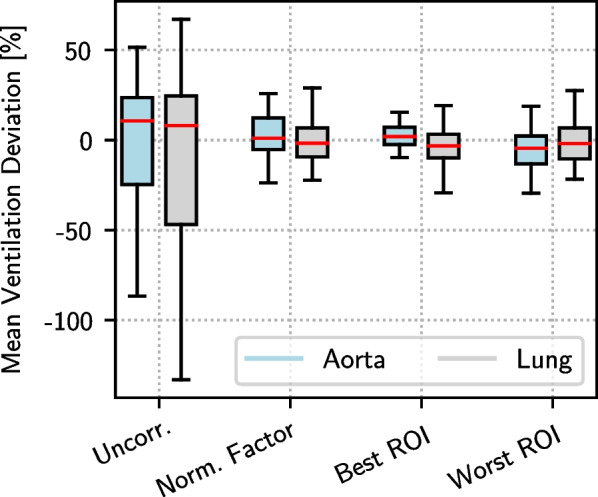
The boxplots of the mean Vw-map deviations. The distributions of the signed mean deviations between each scan and the reference combined for all volunteers are illustrated for both slice positions as boxplots. The whiskers indicate 1.5 times the interquartile range (IQR). Outliers are not shown

This is also confirmed by the Wilcoxon signed rank test. All $p\,$-values indicated a significant improvement at $\alpha \, =\, 0.05$ and are shown in Table 5. Looking at the slice position dependency of the results, there are, except for Volunteers 2, 3 and 4, only minor differences observable between the $\delta _{V}$ of the aorta and the $\delta _{V}$ of the lung slices ranging from 0.5 to $10.9\%$ using the normalization factor. This is also reflected in the median volunteer $\delta _{V}$. However, for both considered ROIs, the normalization performance strongly varies between the slice positions for all volunteers with differences between the $\delta _{V}$ values of up to $19.7\%$ for the best ROI and up to $17.9\%$ for the worst ROI. Slightly better results were obtained for the normalization of the aorta slice compared to the lung slice for all three approaches. This also coincides with the observation that the overall unnormalized reproducibility of the results is slightly worse for the lung slices than for the aorta slices. Figure 7 displays the distributions of the deviations subdivided into aorta and lung, confirming the results from the absolute deviations in Table 4. Combining the Vw-maps of all scans regardless of the slice position, the best ROI leads to the lowest absolute deviation of all three methods and is significantly better than the worst ROI and the normalization factor at $\alpha \, =\, 5\%$. No significant differences between the performances of the factor-based and the normalization using the worst ROI were found. Considering only the Vw-maps of the aorta slices, the best ROI shows significantly lower deviations than the factor-based normalization and the worst ROI, while for the lung slice there was no statistical difference in the performance between all approaches.

**Table 5:** Results of the Wilcoxon signed rank test. The p values of the Wilcoxon signed rank test for the six different pairs are displayed for ventilation. For perfusion, only the comparison between unormalized and normalized using the best perfusion ROI was considered. The ‘*’ indicates statistically significant differences between the compared techniques at =5%

	*p* value					
Pairs	Vw-map			Qw-map		
	Aorta	Lung	Total	Aorta	Lung	Total
Factor versus uncorr.	< 0.01*	< 0.01*	< 0.01*			
Best ROI versus uncorr.	< 0.01*	< 0.01*	< 0.01*	< 0.01*	0.02*	< 0.01*
Worst ROI versus uncorr.	< 0.01*	< 0.01*	< 0.01*			
Best ROI versus Factor	< 0.01*	0.18	0.02*			
Factor versus worst ROI	0.61	0.59	0.71			
Best ROI versus worst ROI	0.03*	0.12	0.01*			

#### Perfusion

The Qw-maps of each scan were normalized using the best perfusion ROI (ROI 5) and the median deviation $\delta _{Q}$ from the reference map for each volunteer and slice position was calculated and compared to the uncorrected maps in Table 6. Using the normalization improved the Qw-map reproducibility in most cases except for the lung slice of Volunteer 1, both slices of Volunteer 4, the lung slice of Volunteers 5 and 8 as well as the aorta slice of Volunteer 9. The differences between the uncorrected and corrected $\delta _{Q}$ varied between $0.1\%$ and $28.0\%$. Comparing the performances between aorta and lung slice for each volunteer, differences between $\delta _{Q}$ of up to $12\%$ were observable for the normalized maps and up to $19.8\%$ for the unnormalized maps. The reduction factor of about 2 between normalized and unnormalized maps was proven to be statistically significant by the Wilcoxon signed rank test (Table 5) for both slice positions as well as the maps of the combined scans.

**Table 6:** The median $\delta _{Q}$ values are listed in % for each volunteer and slice position. The uncorrected deviations are compared to the normalized deviations using the best perfusion ROI (ROI 5). The median volunteer $\varvec{\delta }_{Q}$ for both slices and the combined scans are also presented.

	Median $\varvec{\delta }_{Q}$ over scans in %			
Volunteers	Uncorrected		Best large ROI	
	Aorta	Lung	Aorta	Lung
Vol 1	11.1	4.7	7.2	13.2
Vol 2	8.7	28.5	3.0	7.4
Vol 3	9.1	6.5	5.2	6.0
Vol 4	11.0	3.7	26.5	31.7
Vol 5	16.5	8.0	3.8	9.6
Vol 6	17.1	19.0	7.5	4.8
Vol 7	10.3	10.3	2.1	7.8
Vol 8	9.8	4.5	1.5	4.6
Vol 9	5.3	14.1	18.0	6.0
Vol 10	15.8	9.4	3.6	3.0
Median	11.0	8.8	4.9	5.7
All Scans Median	10.2		5.3	

## Discussion

The feasibility of NuFD MRI at a 0.35 T MR-Linac was evaluated for ten healthy volunteers that underwent repeated scans using normal, deeper and shallower breathing at two different coronal slice positions. It was shown that differences in the breathing amplitude lead to differences in the ventilation-weighted maps, which made the introduction of two normalization strategies necessary. One strategy utilizes the linear relationship between the average lung signal and the diaphragm position to define a scaling factor that corrects for differences in the diaphragm amplitude between the reference scan and the scan that should be normalized. The second strategy is based on the normalization of the ventilation-weighted maps themselves rather than the ventilation signal by selecting a ROI and dividing the ventilation map pixel-wise by the mean value of the ventilation map within this ROI. Even though the perfusion-weighted maps are generally more reproducible due to the robustness of the physiological process, the ROI-based method was also used to normalize the perfusion maps.

Both the scaling-factor-based and the ROI-based normalization strategy show reasonable results for ventilation where the median $\delta _{V}$ was reduced from about $30\%$ with no correction to below $10\%$ for all investigated correction methods and without a considerable difference between different slice positions. For perfusion, using the ROI-based approach, the uncorrected deviations of about $10\%$ could be further reduced to about $5\%$. Both techniques do not depend on any additional equipment such as spirometers and therefore provide a fast normalization workflow that only requires the manual selection of a ROI around the diaphragm to capture the diaphragm motion for the scaling factor approach or at the chosen location in the Vw- or Qw-map for the the ROI-based approach. The idea of fitting the line profile in the former case and extracting the position at the maximum of the derivative of the fit makes use of the fact that the lung parenchyma has a much lower signal than other body parts and therefore causes strong intensity changes at the borders. This makes it a simple and computationally cheap technique compared to more complex feature tracking algorithms. The reason for using the diaphragm motion itself and not the signal of the diaphragm or the 2D lung area as proposed in [37] was to be less dependent on the quality of the image registration and difficulties with motion in and out of the scanned slice. Another advantage of using the presented approaches rather than normalizing the signal itself is that possible global changes in parts of the lung from one scan to another can still be observed, which makes the normalized NuFD suitable for longitudinal studies, such as radiotherapy treatment response monitoring of lung cancer patients.

Even though the results for different slice positions are fairly similar, comparing the volunteers revealed some performance differences for both techniques. Considering the potential integration into longitudinal studies, these robustness aspects have to be looked at in order to decide whether one approach might be better than the other, although there is a statistically significant advantage of using the best ROI. In the case of the scaling factor, one potential reason for these differences between volunteers are possible signal drifts that can cause an underestimation of the signal amplitude (see denominator in Eq. 1) which would lead to an overestimated corrected signal. Since all pixels are multiplied by the scaling factor for both methods, relative differences in the lung are not changed as displayed for the IB Vw-maps in Fig. 6. Additionally, the quality of the image registration plays an important role as it can not only influence the signal amplitude, but also the Vw- and Qw-maps as a consequence of misaligned lung structures. Hence both normalization strategies are affected. Due to differences in the structures visible in the selected slices and the overall intensity, it is reasonable to assume that the registration performance differs for each volunteer. Even though the quantitative influence of the image registration algorithm needs to be further investigated, care was taken during image registration. Visually unsatisfying registration results were re-evaluated and the corresponding scans re-registered with specifically optimized registration parameters. Since the focus of this study was on the analysis of feasibility and reproducibility of the NuFD and the introduced normalization techniques based on relative and not absolute quantitative values, only macroscopic differences in lung density and blood flow were of interest instead of small scale registration differences. The image registration might therefore influence absolute ventilation and perfusion map values, but have limited impact on the main findings of this study in terms of normalization and longitudinal reproducibility.

Another point that needs to be taken into account and concerns the general NuFD workflow is the manual segmentation. According to Willers et al. [46], inter- and intra-observer differences can occur for human observers. In our study this might translate in slight changes in the estimation of the mean value of the Vw- and Qw-maps, but is not expected to have a large impact. However, evaluating patients suffering from COPD and/or lung cancer, differences in the segmentation of the diseased areas has not only a more prominent impact on the mean pixel content of the Vw- and Qw-maps, but also the average lung signal and therefore the normalization factor. This makes it reasonable to potentially consider deep learning-based segmentation approaches for further evaluation.

Even though both approaches are easily realizable in most cases, there are also some limitations. The scaling factor-based normalization requires the tracking of the diaphragm motion. In patients suffering from uni- or bilateral diaphragmatic paralysis, the contraction of the lung is more or less performed by the thorax, namely the accessory muscles of inspiration [47]. In this case, the scaling factor approach would not be practicable and thus the ROI-based normalization would have to be applied. On the other hand, the ROI-based approach reveals not only a slight location and size dependency, but also in order to be able to see possible global changes in specific lung areas, the ROI needs to be positioned on a healthy part of the lung, which are not affected by irradiation in a longitudinal study. Normally, the $12\times 12$ pixels ROI can be easily fitted into the lungs of lung cancer patients, but in special cases, it might be required to reduce the ROI size and to choose a position, which might not coincide with the best location at the lower left lobe and therefore degrades the overall normalization performance. In case of the perfusion, this might in some cases even lead to worse results using normalization compared to the uncorrected scans. However, in patients with severe COPD or CF, where either already the whole lung is affected or the lung function in a formerly healthy lung region worsens over the course of a longitudinal study, this can pose problems and therefore requires future tests to evaluate the applicability of this approach.

The utilization of different evaluation metrics and the novelty of the $0.35\, \textrm{T}$ MR-Linac allows only a limited comparison of the presented study with previously published studies. Lederlin et al. [48] reported a good reproducibility with average differences between ventilation maps of about $6\%$ and between perfusion maps of about $3\%$ obtained $24\, \textrm{h}$ apart in healthy volunteers at a diagnostic $1.5\, \textrm{T}$ MR-scanner using the original FD technique. One major limitation of [48] is that scans were acquired only in normal breathing, which could also explain the differences in mean deviations compared to the reported uncorrected deviations in this study. Similarly, Pöhler et al. [49] investigated the repeatability of ventilation and perfusion parameters derived from the PREFUL technique in healthy volunteers and COPD patients between two normal breathing scans acquired also at a $1.5\, \textrm{T}$ MR-scanner. No significant differences were found between the two acquired scans for the investigated ventilation and perfusion parameters. The study conducted by Voskrebenzev et al. [37] on ventilation reproducibility assessment using a lung area-based and a spirometry-based normalization approach with FD-MRI considered normal and deep breathing scans as well as fixed frequency breathing and chose a more quantitative evaluation approach by calculating the fractional ventilation and the coefficient of variation. Similarly to this study, they found a strong dependence of the ventilation on the breathing amplitude and improved reproducibility by using a normalization strategy with an inter-volunteer coefficient of variation reduction from 0.23 (uncorrected) to 0.12 (normalized).

Generally, the NuFD is not only feasible, but also integrateable into the radiation therapy workflow at a 0.35 T MR-Linac due to the short acquisition time of about 1 min and the lack of contrast agents, respiratory triggering or patient compliance without prolonging treatments. Both normalization strategies improve the reproducibility and comparability of Vw- and Qw-maps in repeated scans.

## Conclusions

In this work, the feasibility of NuFD as a non-contrast enhanced functional lung MRI method to assess ventilation and perfusion has been successfully demonstrated for a 0.35 T MR-Linac using an optimized 2D bSSFP sequence. In order to improve the reproducibility of the ventilation- and perfusion-weighted maps, two normalization techniques have been introduced and tested in a study with ten healthy volunteers, undergoing repeated scans at two different coronal slice positions and utilizing different breathing patterns. Both normalization strategies, the diaphragm amplitude scaling factor and the ROI-based approach, are able to correct for shallow and deep breathing. Averaged over the ten volunteers, median absolute deviations of $9.1\%$ for the normalization factor-based and $5.7\%$/$8.6\%$ for the best/worst ROI-based approach were achieved for ventilation, which shows a clear reduction compared to the deviations of the uncorrected scans of $29.5\%$. Even though perfusion is in general a more regular and reproducible physiological process, using the best perfusion ROI further improved the reproducibility of the perfusion maps from 10.2 to $5.3\%$.

## Supplementary Information

**Additional file 1**: The linear diaphragm position and lung signal correlation. The correlation between the relative diaphragm position and the average lung ventilation signal of each frame are exemplarily shown for a normal breathing scan of Volunteer 4 for aorta (A) and lung slice (B), deep breathing scan of Volunteer 2 (aorta (C) and lung (D)) as well as the shallow breathing scans of Volunteer 8 (aorta (E), lung (F)). The slope of the linear fit function depicted in red finds application in Eq. 1.

**Additional file 2**: Explanatory figure for the diaphragm-based normalization. The mean maxima and minima of the relative diaphragm positions $\overline{x}_{\text {ref,max}}$ and $\overline{x}_{\text {ref,min}}$ are extracted from the corresponding reference scan. The filtered average lung ventilation signal of scan *i*, which should be normalized, is determined and the mean maxima and minima of this signal ($\overline{S}_{i,\text {max}}$, $\overline{S}_{i,\text {min}}$) calculated from the respective peaks. This filtered average lung ventilation signal is also correlated with the relative diaphragm positions of scan *i*. Fitting this correlation allows to extract the slope $\text {d}S_{i}/ \text {d}x_{i}$.

**Additional file 3**: Boxplot comparison of ROI size and location for the Vw-maps. The boxplot for the signed mean deviations for each ROI using the $8\times 8$ pixels square in (A) and the $12\times 12$ pixels square in (B) compared to the uncorrected scans. Here, all scans of all volunteers were combined regardless of the slice position. The whiskers indicate 1.5 times the interquartile range (IQR). Outliers are not shown.

**Additional file 4**: The $\delta _{V}$ values of Volunteer 5 for the uncorrected Vw-maps as well as for the normalized Vw-maps using the diaphragm-based and the best and worst ROI-based normalization. The corresponding maps to these values are shown in Fig. 6.

## Data Availability

The datasets used and/or analysed during the current study are available from the corresponding author on reasonable request.

## Abbreviations

aB: After break
ART: Adaptive radiotherapy
bSSFP: Balanced steady-state free precession
CF: Cystic fibrosis
COPD: chronic obstructive pulmonary disease
CT: Computed tomography
IB: Irregular breathing
IQR: Interquartile range
MRI: Magnetic resonance imaging
NB: Normal breathing
NuFD: Non-uniform Fourier Decomposition
NuFFT: Non-uniform Fourier Transform
Q: Perfusion
Qw: Perfusion-weighted
ROI: Region-of-interest
TE: Echo time
TR: Repetition time
V: Ventilation
Vw: Ventilation-weighted
