# Supplementary figures and images for: Ventilation and perfusion MRI at a 0.35 T MR-Linac: feasibility and reproducibility study
Source: Radiat Oncol. 2023 Apr 3;18:58. doi: 10.1186/s13014-023-02244-1 (PMC10069152; doi:10.1186/s13014-023-02244-1)

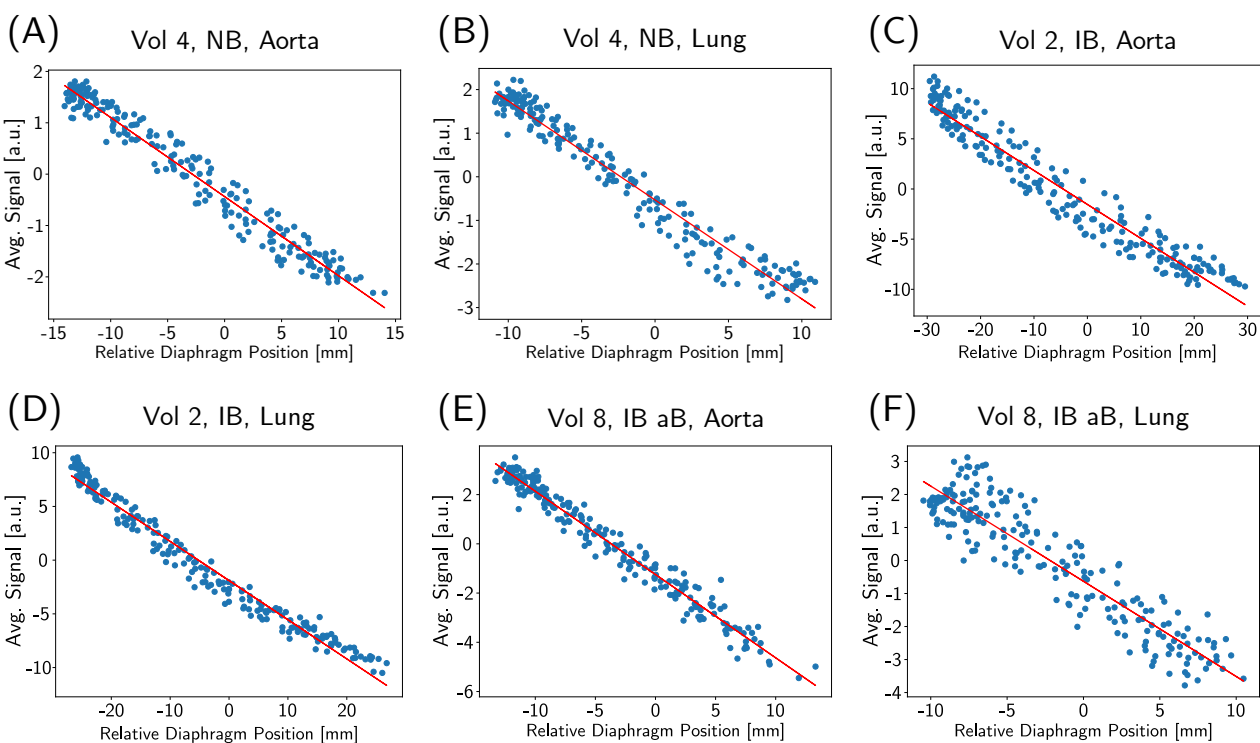

Supplement: Supplementary file 1 — Additional file 1: The linear diaphragm position and lung signal correlation. The correlation between the relative diaphragm position and the average lung ventilation signal of each frame are exemplarily shown for a normal breathing scan of Volunteer 4 for aorta (A) and lung slice (B), deep breathing scan of Volunteer 2 (aorta (C) and lung (D)) as well as the shallow breathing scans of Volunteer 8 (aorta (E), lung (F)). The slope of the linear fit function depicted in red finds application in Eq. 1. [file 13014_2023_2244_MOESM1_ESM.pdf]

(A)

Small ROI

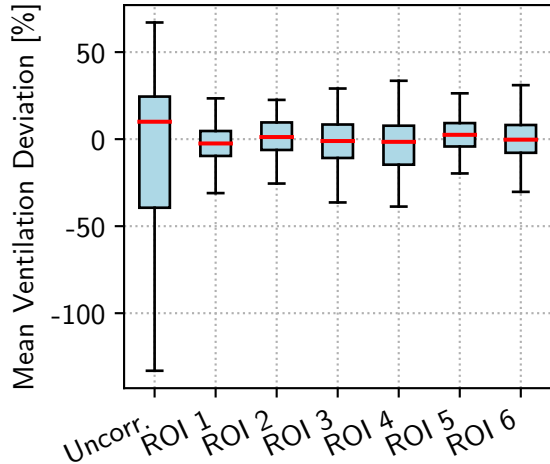

(B)

Large ROI

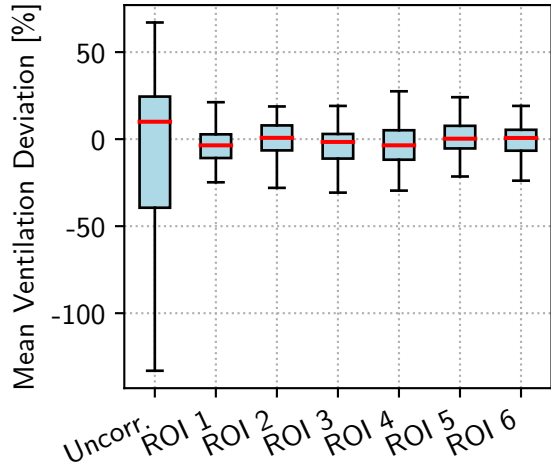

Supplement: Supplementary file 3 — Additional file 3: Boxplot comparison of ROI size and location for the Vw-maps. The boxplot for the signed mean deviations for each ROI using the $8\times 8$8×8 pixels square in (A) and the $12\times 12$12×12 pixels square in (B) compared to the uncorrected scans. Here, all scans of all volunteers were combined regardless of the slice position. The whiskers indicate 1.5 times the interquartile range (IQR). Outliers are not shown. [file 13014_2023_2244_MOESM3_ESM.pdf]
