# Supplementary material for: Ventilation and perfusion MRI at a 0.35 T MR-Linac: feasibility and reproducibility study
Source: Radiat Oncol. 2023 Apr 3;18:58. doi: 10.1186/s13014-023-02244-1 (PMC10069152; doi:10.1186/s13014-023-02244-1)
Supplement: Supplementary file 2 — Additional file 2: Explanatory figure for the diaphragm-based normalization. The mean maxima and minima of the relative diaphragm positions $\overline{x}_{\text {ref,max}}$x¯ref,max and $\overline{x}_{\text {ref,min}}$x¯ref,min are extracted from the corresponding reference scan. The filtered average lung ventilation signal of scan i, which should be normalized, is determined and the mean maxima and minima of this signal ($\overline{S}_{i,\text {max}}$S¯i,max, $\overline{S}_{i,\text {min}}$S¯i,min) calculated from the respective peaks. This filtered average lung ventilation signal is also correlated with the relative diaphragm positions of scan i. Fitting this correlation allows to extract the slope $\text {d}S_{i}/ \text {d}x_{i}$dSi/dxi. [file 13014_2023_2244_MOESM2_ESM.pdf]

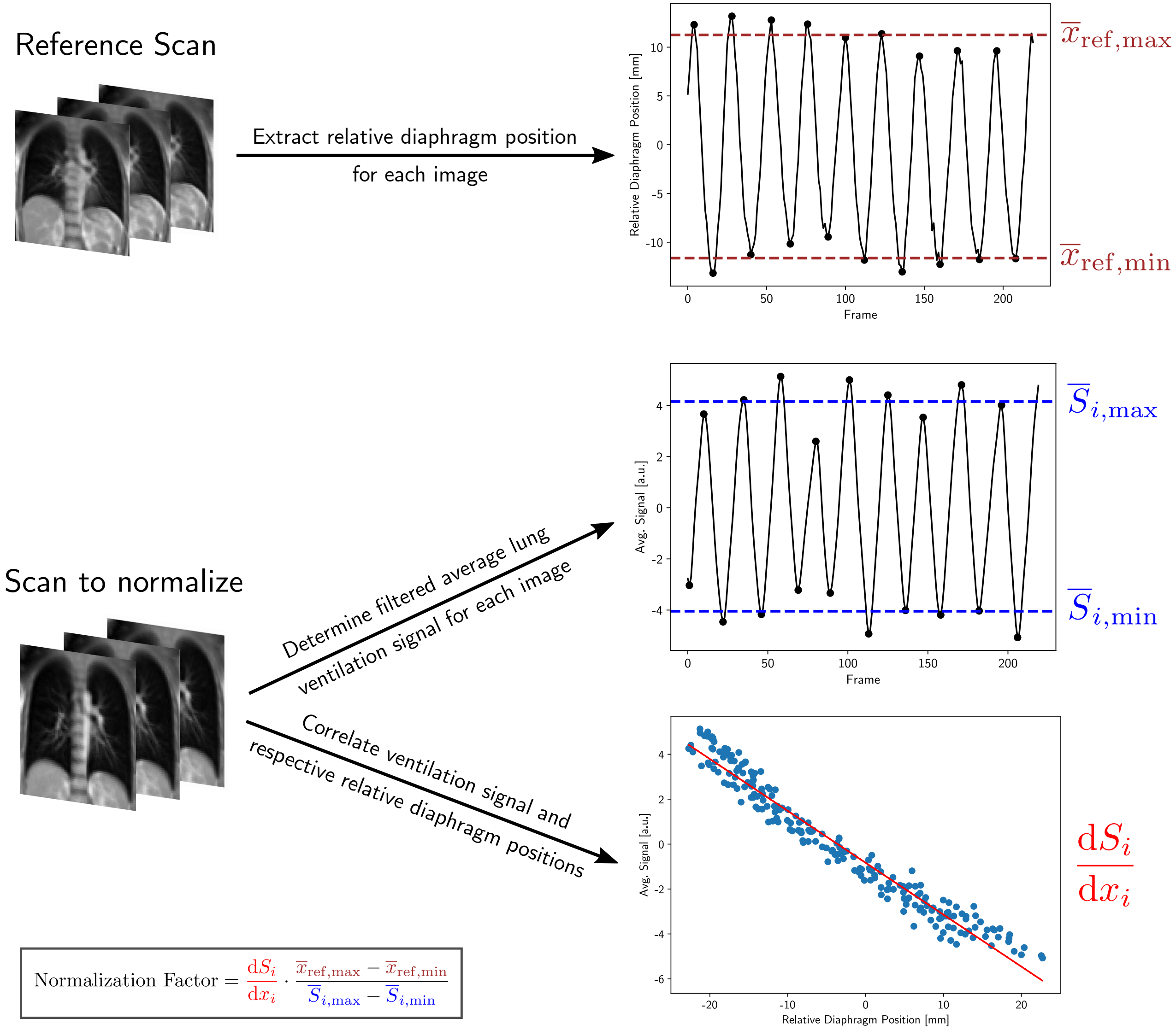

Reference Scan
Extract relative diaphragm position
for each image
Relative Diaphragm Position [mm]
Frame
$\overline{x}_{\mathrm{ref,max}}$
$\overline{x}_{\mathrm{ref,min}}$
Scan to normalize
Determine filtered average lung
ventilation signal for each image
Avg. Signal [a.u.]
Frame
$\overline{S}_{i,\mathrm{max}}$
$\overline{S}_{i,\mathrm{min}}$
Correlate ventilation signal and
respective relative diaphragm positions
Avg. Signal [a.u.]
Relative Diaphragm Position [mm]
$\frac{\mathrm{d}S_i}{\mathrm{d}x_i}$
Normalization Factor $= \frac{\mathrm{d}S_i}{\mathrm{d}x_i} \cdot \frac{\overline{x}_{\mathrm{ref,max}} - \overline{x}_{\mathrm{ref,min}}}{\overline{S}_{i,\mathrm{max}} - \overline{S}_{i,\mathrm{min}}}$
