# Supplementary material for: Ventilation and perfusion MRI at a 0.35 T MR-Linac: feasibility and reproducibility study
Source: Radiat Oncol. 2023 Apr 3;18:58. doi: 10.1186/s13014-023-02244-1 (PMC10069152; doi:10.1186/s13014-023-02244-1)
Supplement: Supplementary file 4 — Additional file 4: The $\delta _{V}$δV values of Volunteer 5 for the uncorrected Vw-maps as well as for the normalized Vw-maps using the diaphragm-based and the best and worst ROI-based normalization. The corresponding maps to these values are shown in Fig. 6. [file 13014_2023_2244_MOESM4_ESM.pdf]

Scans	$\boldsymbol{\delta_V}$ **in %**			
	Uncorrected	Normalization Factor	Best Large ROI	Worst Large ROI
NB 2	23.6	20.4	3.7	2.4
IB	114.9	15.2	5.7	14.5
NB aB	0.43	9.7	5.0	4.4
NB aB 2	24.6	5.1	0.1	5.1
IB aB	23.0	1.5	7.3	5.5
Median	23.6	13.3	5.0	5.1
